# Fibrodysplasia ossificans progressiva (stone man syndrome): a case report

**DOI:** 10.1186/s13256-019-2297-z

**Published:** 2019-12-01

**Authors:** Zakir Ali Shah, Sascha Rausch, Uzma Arif, Bilal El Yafawi

**Affiliations:** 1 Department of Trauma and Orthopedic, Rashid Hospital, Dubai, 4545 United Arab Emirates; 2 Department of Diagnostic Radiology, Sheikh Zayed Hospital, Lahore, Pakistan

**Keywords:** Fibrodysplasia ossificans progressiva, Myositis ossificans progressiva, Stone man syndrome

## Abstract

**Background:**

Fibrodysplasia ossificans progressiva is an ultrarare autosomal dominant disorder and disabling syndrome characterized by postnatal progressive heterotopic ossification of the connective tissue and congenital malformation of the big toes. Fibrodysplasia ossificans progressiva has worldwide prevalence of about 1 in 2 million births. Nearly 90% of patients with fibrodysplasia ossificans progressiva are misdiagnosed and mismanaged and thus undergo unnecessarily interventions. So far, the number of reported existing cases worldwide is about 700. Clinical examination, radiological evaluation, and genetic analysis for mutation of the *ACVR1* gene are considered confirmatory tools for early diagnosis of the disease. Association of fibrodysplasia ossificans progressiva with heterotopic ossification is well documented; however, postsurgical exaggerated response has never been reported previously, to the best of our knowledge.

**Case presentation:**

We report a case of a 10-year-old Pakistani boy brought by his parents to our institution. He had clinical and radiological features of fibrodysplasia ossificans progressive and presented with multiple painful lumps on his back due to hard masses and stiffness of his shoulders, neck, and left hip. He underwent surgical excision of left hip ossification followed by an exaggerated response in ossification with early disability. Radiological examination revealed widespread heterotopic ossification. All of his laboratory blood test results were normal.

**Conclusion:**

Fibrodysplasia ossificans progressiva is a very rare and disabling disorder that, if misdiagnosed, can lead to unnecessary surgical intervention and disastrous results of early disability. We need to spread knowledge to physicians and patients’ family members about the disease, as well as its features for early diagnosis and how to prevent flare-up of the disease to promote better quality of life in these patients.

## Background

Fibrodysplasia ossificans progressiva (FOP) is a very rare disorder with a worldwide prevalence of approximately 1 in 2 million population. The age of onset is mostly in the first two decades of life, and there is no ethnic, racial, gender, or geographic predilection of FOP [1].

FOP is a disorder in which congenital abnormalities of the big toes are associated with progressive heterotopic ossification of the connective tissue structures, including those related to the striated muscles, leading to permanent disability [2, 3].

The development of rapidly growing masses, usually in the neck or paravertebral region, is caused by fibroblastic proliferation and ossification of underlying soft tissues. The soft tissue masses can grow spontaneously, but trauma can aggravate the growth and calcification. FOP is often misdiagnosed, which results in unnecessary biopsies and surgeries that result in exacerbation of disease. Therefore, awareness of the clinical features of FOP by all physicians, surgeons, and pediatricians is essential for early diagnosis [4].

It has recently been found that FOP is caused by heterozygous activating mutations in activin A receptor, type 1 (ACVR1), also known as activin-like kinase 2, which is a bone morphogenetic protein type 1 receptor [5, 6].

## Case presentation

A 10-year-old Pakistani boy presented to our clinic in Pakistan with a 6-month history of pain and tender masses on the back, left arm, and left hip. Pain was insidious in onset and gradually worsened, causing difficulty in walking and decreased range of motion of shoulders and hips.

**Fig. 1:**
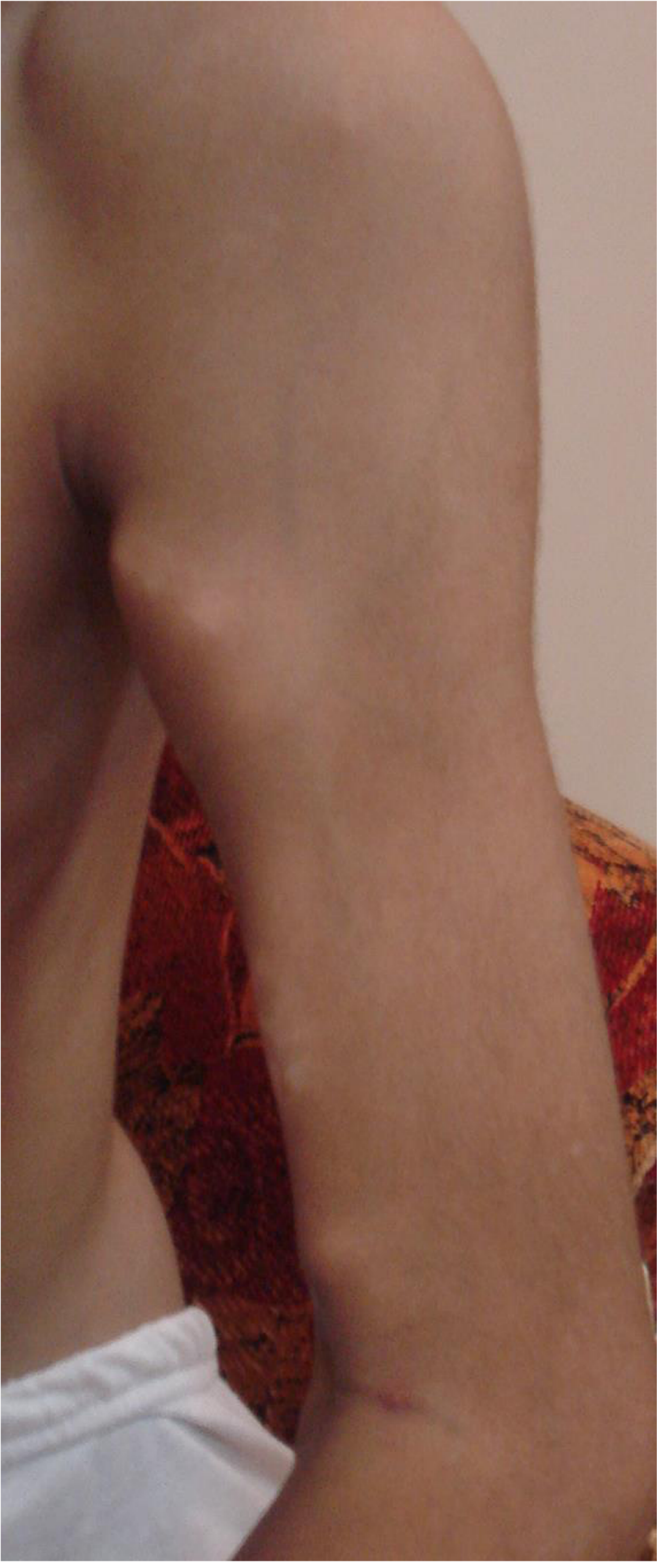
Presence of mass on the left arm and restricted shoulders abduction with a 35-degree angle

On examination, the patient had multiple swellings on his back, right shoulder, left hip, and left knee. Another hard mass was seen on the left arm, near the anterior fold of the armpit, and extending to the whole of the biceps. The mass was painful, but no warmth or inflammation was noted (Fig. 1). Palpation revealed tenderness of all visible masses and stiffness of all abdominal and paraspinal muscles. Abduction of both shoulders was restricted to 35 degrees on the left side and 10 degrees on the right side (Fig. 1).

**Fig. 2:**
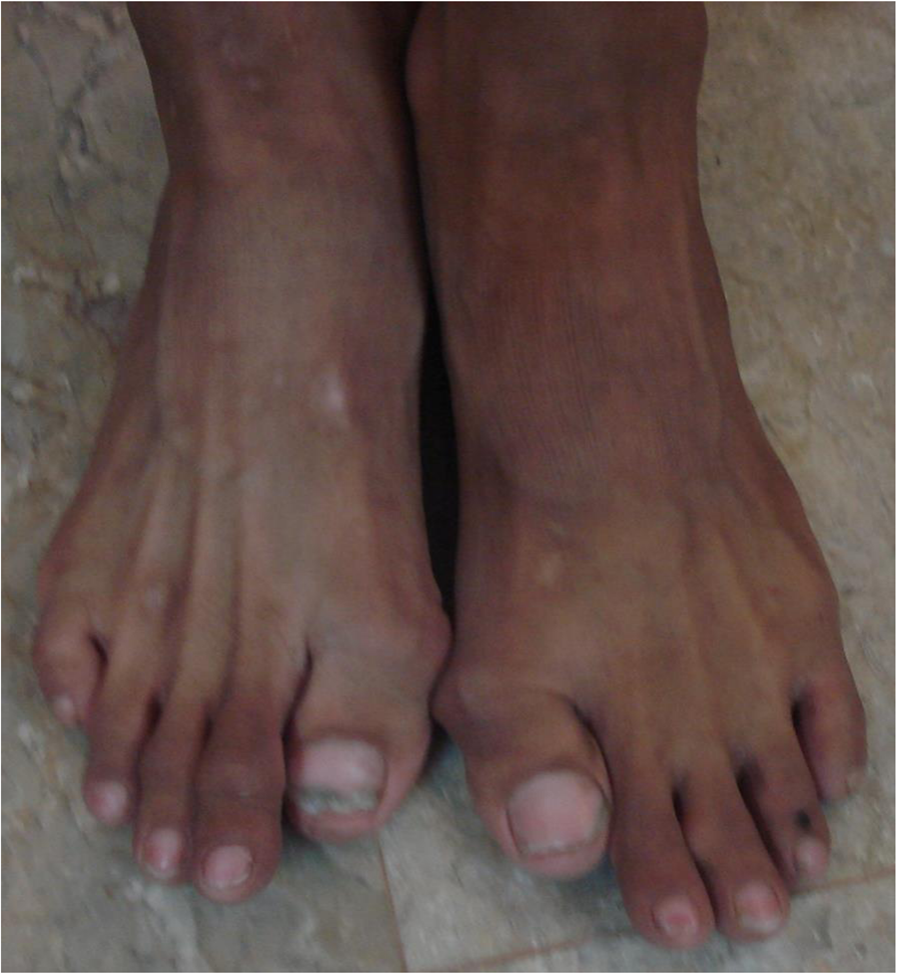
Hallux valgus bilateral: clinical view

The patient had bilateral hallux valgus but no other abnormality of any other toes (Fig. 2).

**Fig. 3:**
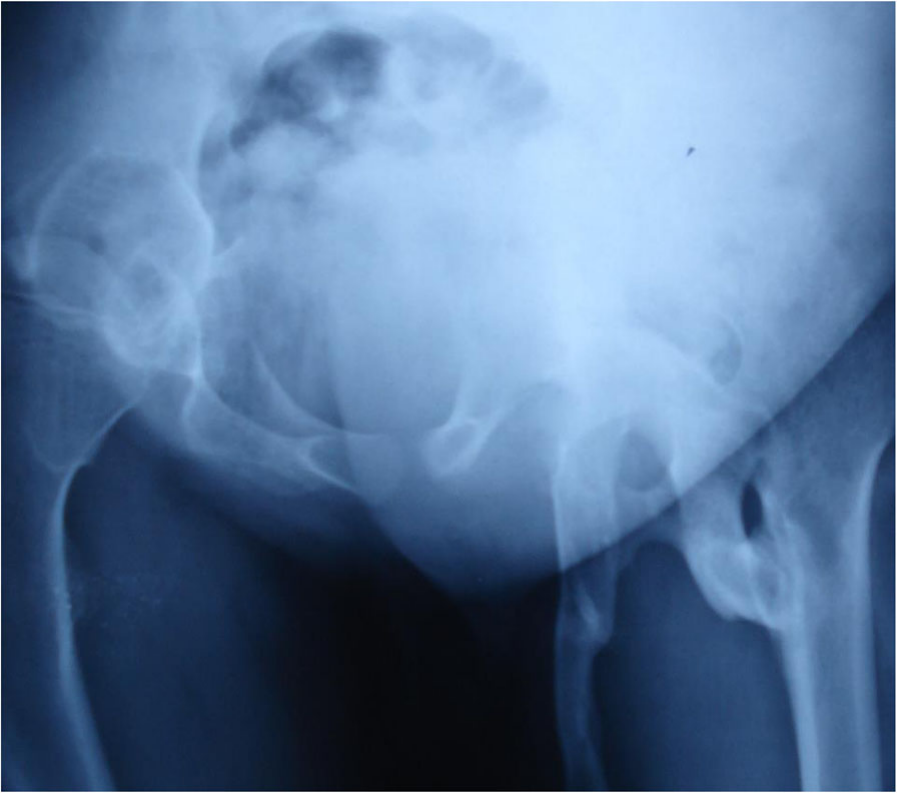
Presurgery: Presence of ossification of right hip and mass demonstrating fusion of left hip joint, broad femur neck, and ossification columns between pelvic bones and femur

He was not able to walk and squat in normal posture and had severe restricted range of motion of the left hip (Fig. 3).

No appreciable lymphadenopathy was noted. The boy’s parents did not show any similar abnormalities in their physical examination.

**Fig. 4:**
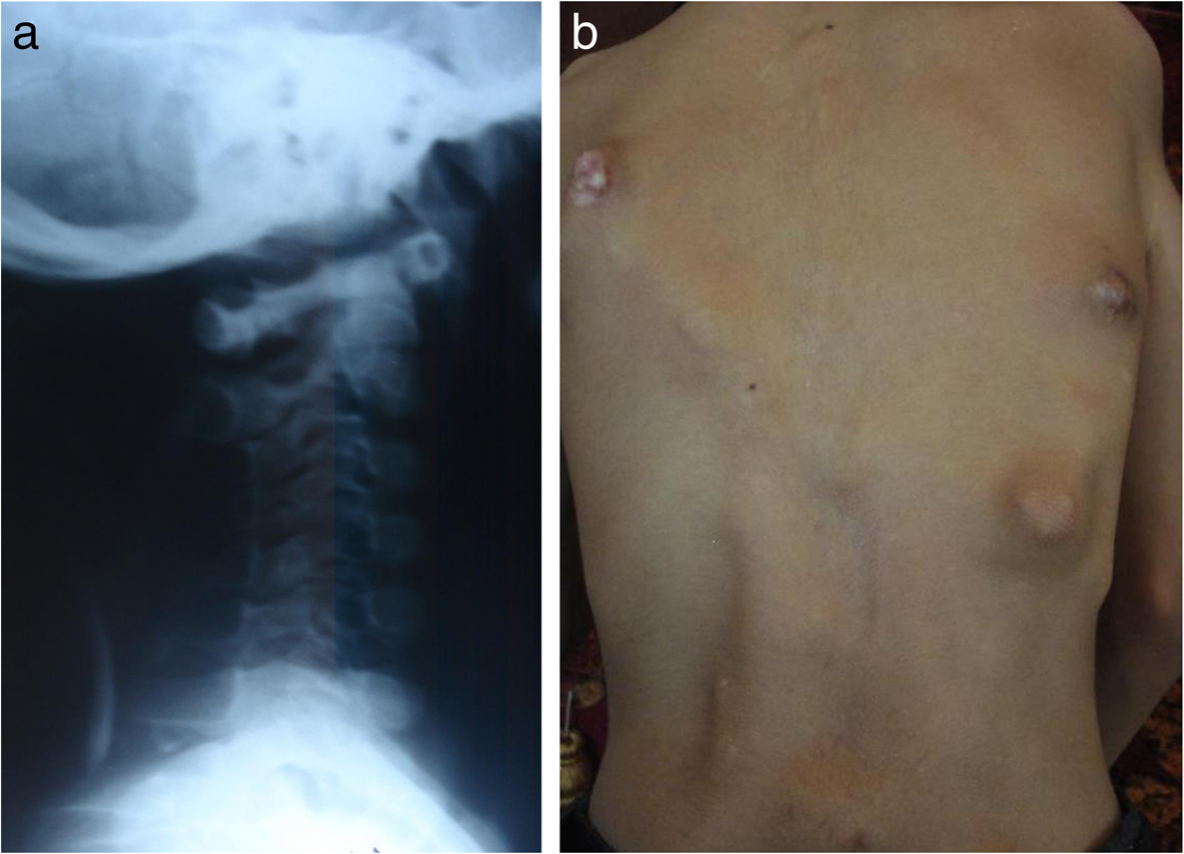
**a** Presence of mass behind cervical spine. **b** Presence of multiple masses on back

**Fig. 5:**
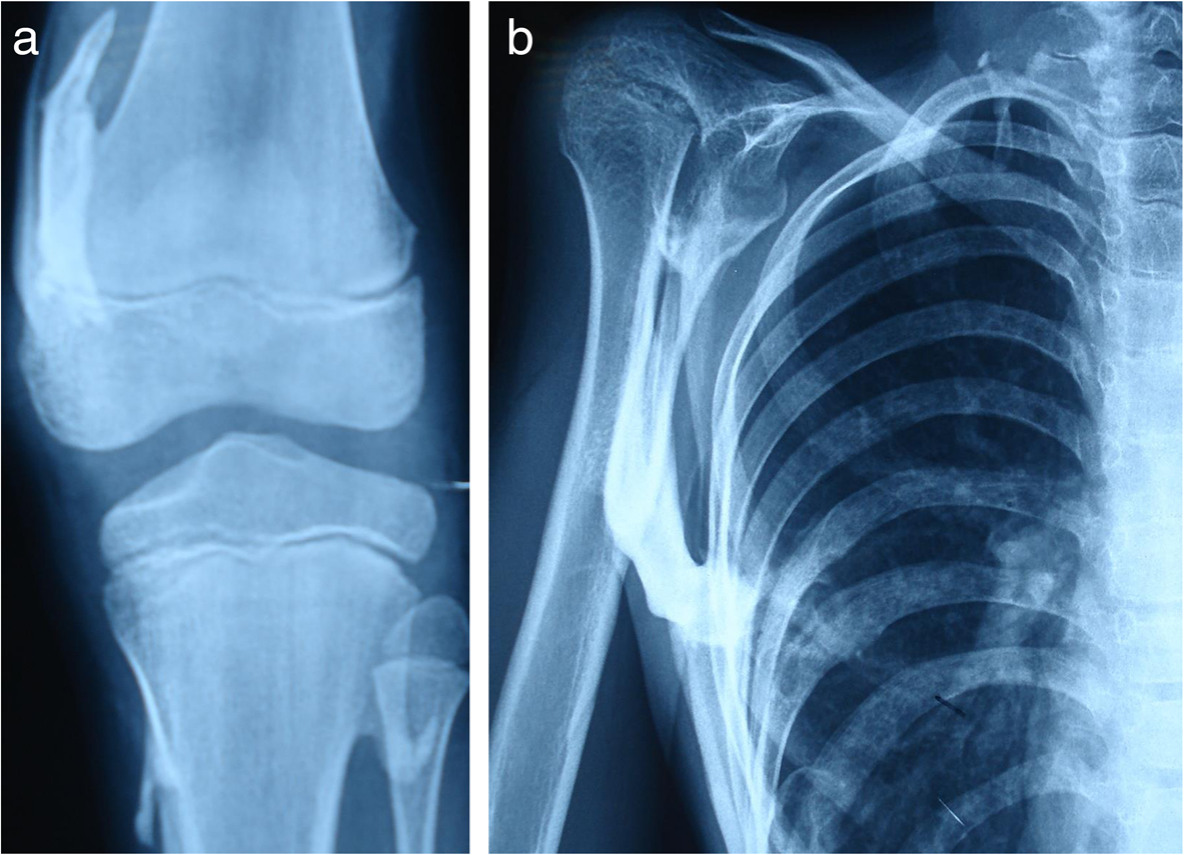
**a** Presence of ossification left distal femur and proximal tibia. **b** Ossification involving right shoulder

Results of laboratory studies were normal. Genetic analysis testing could not be performed because of the parents’ financial issues. Conventional radiographs showed heterotopic ossification involving the spine, neck, shoulders, hips, and right knee (Figs. 4 and 5).

No history of local trauma was found at the beginning of the disease. The patient had no siblings and no family history of any disease.

**Fig. 6:**
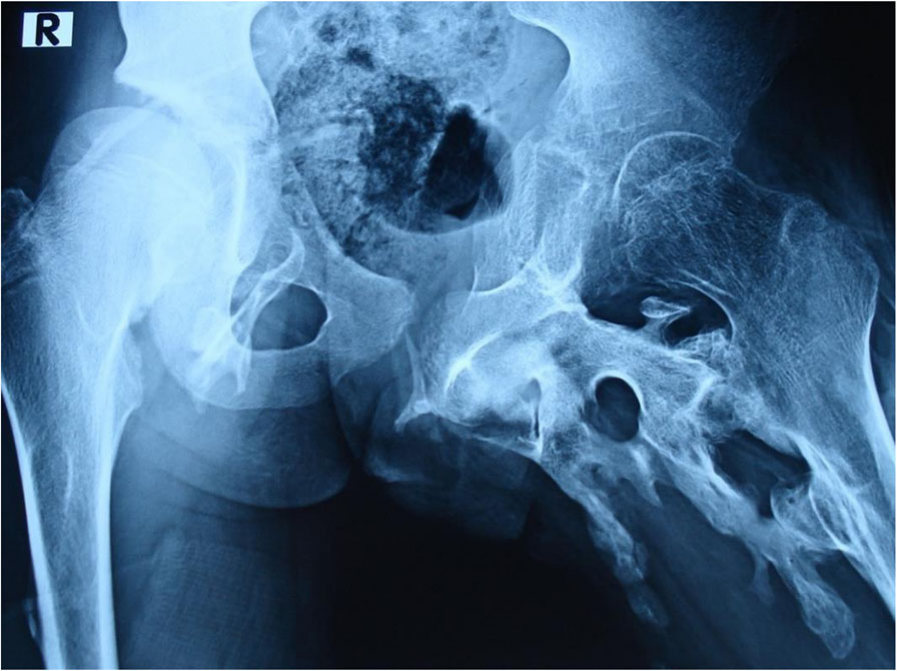
Follow up x-ray after surgery/excision: Presence of ossification of right hip comparable with preoperative x-ray; however, excessive flare-up of ossification of left hip is seen

Regarding the patient’s past history, his parents reported surgical intervention and excision of left hip ossification followed by temporary improvement in range of motion of the left hip. Regular follow-up was done at 2-month intervals, and follow-up x-ray showed exacerbation (flare-up) of ossification and again severe restricted range of motion of the left hip (Fig. 6).

The patient was initiated on symptomatic treatment, and his family was educated about the disease. Counseling was done, and prevention of trivial trauma was advised. Recently, in follow-up, it was noticed that his quality of life was improved over a previous visit. The patient will be followed clinically and radiologically.

## Discussion

In 1692, FOP was first described by Guy Patin in a young patient who “turned to wood” [7].

Myositis ossificans is a very rare disease characterized by heterotopic ossification formation, typically involving muscles, tendons, ligaments, fascia, and aponeurosis. FOP is a rare, hereditary, progressive connective tissue disorder characterized by congenital malformation of the great toes; progressive ossificans occurs mainly in the neck, chest, and back [8, 9].

In our patient’s case, his parents reported that he had had hallux valgus since birth [9, 10]. The initial symptoms of FOP are painful and hard soft tissue swellings over the affected muscles that lead to ossification. It usually occurs from birth to the second decade of life, following spontaneous or trauma-induced flare-ups [11]. Heterotopic ossification usually begins in the cervical paraspinal muscles and later spreads from axial to appendicular, from cranial to caudal, and from proximal to distal sites. Scoliosis is a common finding because of asymmetric heterotopic bones connecting the trunk and pelvis [12]. Fusion of ossicles of the ear leads to conductive hearing loss, which is a common feature associated with this condition [6, 13, 14]. Progressive episodes of heterotopic ossification lead to ankylosis of all major joints of the axial and appendicular skeleton, rendering movement impossible. In the second decade of life, mostly patients with FOP are confined to bed or wheelchair [6, 15]. The most common cause of death in FOP is cardiopulmonary failure resulting from thoracic insufficiency syndrome [16].

FOP diagnosis is clinical, and it is usually made on the basis of the presence of three major criteria [6, 14]: congenital malformation of the great toes, progressive heterotopic endochondral ossification, and progression of the disease in well-defined anatomical and temporal patterns. Laboratory tests may show a discreet increase of the erythrocyte sedimentation rate during the flare-ups. Genetic analysis for *ACVR1* gene mutation is a confirmatory test. Imaging examinations such as radiography and computed tomography show the heterotopic bones and are useful to confirm the diagnosis.

FOP should be differentiated from other similar conditions, including progressive osseous heteroplasia, Albright hereditary osteodystrophy, osteoma cutis, ankylosing spondylitis, Still disease, Klippel-Feil-syndrome, brachydactyly, juvenile bunions, sarcoma, and desmoid tumor [9, 14].

Treatment of FOP is administered using a multidisciplinary approach based on injury prevention, conservative use of analgesics, and surgery.

Surgical excision is considered when excessive pain, joint limitation, or nerve compression is present. Surgery generally is advised when myositis ossificans is ripe, identified by a higher bone density in x-ray findings and normal erythrocyte sedimentation rate and alkaline phosphatase level.

Physical rehabilitation should be focused on enhancing activities of daily living through approaches that avoid passive range of motion that could lead to disease flare-ups [6, 8]. Flare-ups of FOP may occur spontaneously or be precipitated by trauma, such as intramuscular injections, including vaccines and muscle biopsy. Moreover, in routine dental care, overstretching of the jaw and intramuscular local anesthetic injections also should be avoided. Patients with FOP may have an additional risk of flare-ups after influenza-like illness. Thus, a subcutaneous influenza vaccine could help these patients, particularly those who have severe restrictive disease of the chest wall and are at a greater risk of presenting with complications of respiratory infections, which are a frequent cause of death [6, 11]. Drug treatment of our patient was based on the current treatment guidelines published by Kaplan *et al.* [17]. Corticosteroids are advised as the first line of treatment at the beginning of flare-ups. The use of corticosteroids should be restricted to treatment of flare-ups that affect major joints, the jaw, or the submandibular area. Corticosteroids should not be used for symptomatic treatment of flare-ups that involve the back, neck, or trunk, owing to the long duration and recurring nature of these flare-ups and the difficulty in assessing the true onset of such flare-ups [18]. When prednisone is discontinued, a nonsteroidal anti-inflammatory drug or a Cox-2 inhibitor (in conjunction with a leukotriene inhibitor) may be used symptomatically for the duration of the flare-up [19]. None of these drugs avoided the progression of the disease in our patient.

## Conclusion

FOP is a rare and disabling disorder that still does not have an effective treatment that can cure it or stop its progression. Mainly, physicians, surgeons, and patients and their families should be educated about the disease, and proper counseling of families should be provided.

Symptomatic treatment with drugs is advised; the best approach is still considered early diagnosis and prevention of injury to patients to help them avoid flare-ups. Surgeons should avoid unnecessary excision of heterotopic ossifications until they are fully ripe and surgery is strongly indicated.

## Data Availability

All of the data appear within this report.

## Abbreviations

ACVR1: Activin A receptor, type1
FOP: Fibrodysplasia ossificans progressiva
